# Binding site of ABC transporter homology models confirmed by ABCB1 crystal structure

**DOI:** 10.1186/1742-4682-6-20

**Published:** 2009-09-04

**Authors:** Aina W Ravna, Ingebrigt Sylte, Georg Sager

**Affiliations:** 1 Department of Medical Pharmacology and Toxicology, Institute of Medical Biology, Faculty of Health Sciences, University of Tromsø, N-9037 Tromsø, Norway

## Abstract

The human ATP-binding cassette (ABC) transporters ABCB1, ABCC4 and ABCC5 are involved in resistance to chemotherapeutic agents. Here we present molecular models of ABCB1, ABCC4 and ABCC5 by homology based on a wide open inward-facing conformation of *Escherichia coli *MsbA, which were constructed in order to elucidate differences in the electrostatic and molecular features of their drug recognition conformations. As a quality assurance of the methodology, the ABCB1 model was compared to an ABCB1 X-ray crystal structure, and with published cross-linking and site directed mutagenesis data of ABCB1. Amino acids Ile306 (TMH5), Ile340 (TMH6), Phe343 (TMH6), Phe728 (TMH7), and Val982 (TMH12), form a putative substrate recognition site in the ABCB1 model, which is confirmed by both the ABCB1 X-ray crystal structure and the site-directed mutagenesis studies. The ABCB1, ABCC4 and ABCC5 models display distinct differences in the electrostatic properties of their drug recognition sites.

## Introduction

The human ATP-binding cassette (ABC) transporters ABCB1, ABCC4 and ABCC5 belong to the ABC superfamily, a subgroup of Primary active transporters [1]. The transporters in the ABC superfamily are structurally related membrane proteins that have a common intracellular motif that exhibits ATPase activity. This motif cleaves ATP's terminal phosphate to energize the transport of molecules from regions of low concentration to regions of high concentration [1-3]. Since ABC genes are highly conserved between species, it is likely that most of these genes have been present since the beginning of eukaryotic evolution [4].

**Figure 1:**
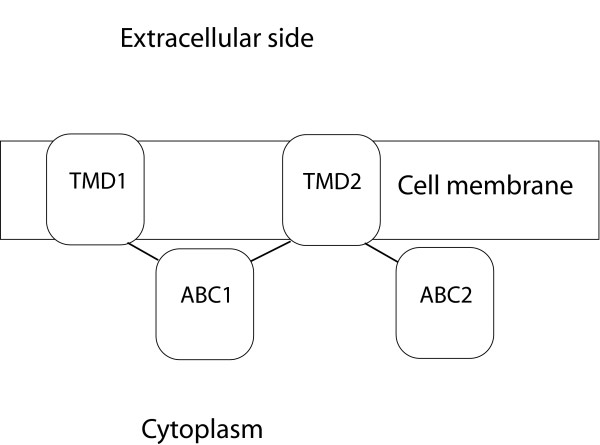
**Overall domain topology of ABCB1, ABCC4 and ABCC5**.

The overall topology of ABCB1, ABCC4 and ABCC5 is divided into transmembrane domain 1 (TMD1) - nucleotide-binding domain 1 (NBD1) - TMD2 - NBD2 (Figure 1). The Walker A, or phosphate binding loop (P-loop), and Walker B motifs, are localized in the NBDs, while the TMDs contribute to the substrate translocation events (recognition, translocation and release). ABCB1, ABCC4 and ABCC5 are exporters, pumping substrates out of the cell.

Transporters have drug recognition sites that make them specific for particular substrates, and drugs may interact with these recognition sites and either inhibit the transporter or act as substrates. Experimental studies have shown that ABCB1 transports cationic amphiphilic and lipophilic substrates [5-8], while ABCC4 and ABCC5 transport organic anions [9]. Both ABCC4 and ABCC5 transport cAMP and cGMP, however, with differences in their kinetic parameters; ABCC4 with a preference for cAMP and ABCC5 with a preference for cGMP [9,10].

When chemotherapeutic agents are expelled from cancer cells as substrates of ABCB1, ABCC4 or ABCC5, the result is multidrug resistance. In order to overcome multidrug resistance, development of inhibitors of drug efflux transporters has been sought for use as supplement to drug therapy [11]. However, clinical trials of potential anti-MDR agents have been disappointing due to adverse effects *in vivo *of agents being very effective *in vitro*. Even if there is a long time since Victor Ling described MDR, (i.e. ABCB1) [12], very little is known about subtype selective recognition and binding of ABC proteins. Structural insight into their mode of ligand interaction and functional mechanisms will be an important contribution to pinpoint potential drug targets and to design putative inhibitors. Recent papers report a considerable difference in substrate specificity of ABCC4 and ABCC5 [9], including various chemotherapeutic agents [13], and with potential impact on reversal of MDR [14]. Elucidating the molecular aspects of ligand interactions with ABCB1, ABCC4 or ABCC5 may therefore aid in the design of therapeutic agents that can help to overcome multidrug resistance.

We have previously constructed molecular models of ABCB1 [15], ABCC4 [16] and ABCC5 [17] based on the *Staphylococcus aureus *ABC transporter Sav1866, which has been crystallized in an outward-facing ATP-bound state [18]. In this study, we present molecular models of ABCB1, ABCC4 and ABCC5 based on a wide open inward-facing conformation of *Escherichia coli *MsbA [19]. Since the molecular modelling was carried out before the X-ray crystal structure of the *Mus musculus *ABCB1 in a drug-bound conformation was published [20], we got a unique opportunity to test our methodology, molecular modelling by homology, and the quality of the ABCB1 model, when the crystal structure was published. Since we wanted to elucidate differences in the electrostatic and molecular features of the drug recognition conformation of these transporters, the wide open conformation of the MsbA template [19] was of particular interest. The electrostatic potential surfaces (EPS) of the models were calculated, and the models were compared to the X-ray crystal structure of the *Mus musculus *ABCB1 [20], and with published cross-linking and site directed mutagenesis data on ABCB1 [21-35].

## Computational methods

### Software

Version 3.4-9b of the Internal Coordinate Mechanics (ICM) program [36] was used for homology modelling, model refinements and electrostatic calculations. The AMBER program package version 8.0 [37] was used for molecular mechanics energy minimization.

### Alignment

A multiple sequence alignment of (SWISS-PROT accession numbers are given in brackets) human ABCB1 (P08183), human ABCC4 (O15439), human ABCC5 (O15440), human ABCC11 (Q9BX80), *Escherichia coli *MsbA (P60752) and *Vibrio cholerae *MsbA (Q9KQW9), obtained using T-COFFEE [38], Version 4.71 available at the Le Centre national de la recherche scientifique website , was used as a basis for the homology modelling module of ICM program [36]. ABCC11 was included in the alignment because it is closely related to ABCC5 phylogenetically [15], and its inclusion may strengthen the alignment. The alignment was adjusted for sporadic gaps in the TMH segments, and for secondary structure predictions defining the boundaries of the TMHs using the PredictProtein server for sequence analysis and structure prediction [39], and SWISS-PROT [40].

**Figure 2:**
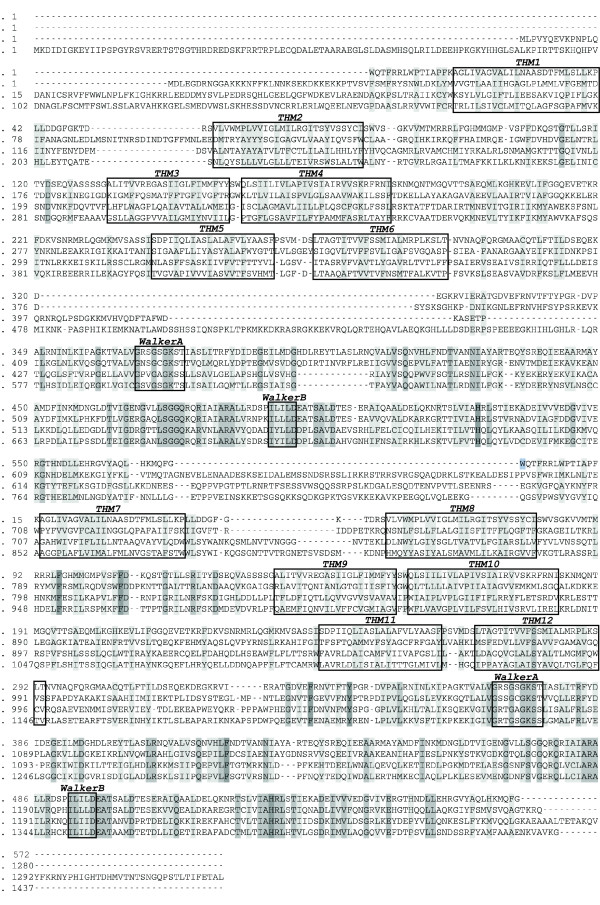
**Alignment of *Escherichia coli *MsbA, human ABCB1, human ABCC4 and human ABCC5 used as input alignment for the ICM homology modelling module**. TMHs, Walker A motifs and Walker B motifs are indicated as boxes.

The alignment of human ABCB1 and *Escherichia coli *MsbA was compared to previously published alignments of human ABCB1 and *Escherichia coli *MsbA [19,41], and it was observed that in our alignment, the ABCB1 sequence was shifted 2 positions to the left relative to the E. coli MsbA sequence in the alignment of TMH2, and 1 position the left relative to the E. coli MsbA sequence in the alignment of TMH6, as compared to the previously published alignments of human ABCB1 and *Escherichia coli *MsbA [19,41]. Thus, 3 alignments were used to construct 3 ABCB1 models, 1 model with our original alignment, 1 model with TMH2 adjusted to correspond to the previously published alignments of human ABCB1 and *Escherichia coli *MsbA [19,41], and 1 model with both TMH2 and TMH6 adjusted, thus using the same alignment as the previously published alignments of human ABCB1 and *Escherichia coli *MsbA [19,41]. The alignment of *Escherichia coli *MsbA, human ABCB1, human ABCC4 and human ABCC5 used for the homology modelling procedure, with TMH2 adjusted to correspond to the previously published alignments of human ABCB1 and *Escherichia coli *MsbA [19,41], is shown in Figure 2. For illustrative purposes, only the sequences of the template and the 3 target proteins ABCB1, ABCC4 and ABCC5 are shown.

### Homology modelling

A full atom version of the open inward facing *Escherichia coli *MsbA X-ray crystal structure (PDB code: 3B5W[19]) was kindly provided by Geoffery Chang and used as a template in the construction of the homology models of ABCB1, ABCC4 and ABCC5. The ICM program constructs the molecular model by homology from core sections defined by the average of Cα atom positions in conserved regions. Loops were searched for within several thousand structures in the PDB databank [42] and matched in regard to sequence similarity and sterical interactions with the surroundings of the model, and the best-fitting loop was selected based on calculating the maps around the loops and scoring of their relative energies. The segment connecting NBD1 and TMD2 was also included in the loop search procedure.

### Calculations

The ABCB1, ABCC4 and ABCC5 models were refined by globally optimizing side-chain positions and annealing of the backbone using the RefineModel macro of ICM. The macro was comprised of (1) a side-chain conformational sampling using 'Montecarlo fast' [43], (2) 5 iterative annealings of the backbone with tethers (harmonic restraints pulling an atom in the model to a static point in space represented by a corresponding atom in the template), and (3) a second side-chain conformational sampling using 'Montecarlo fast'. 'Montecarlo fast' samples conformational space of a molecule with the ICM global optimization procedure, and its iterations consist of a random move followed by a local energy minimization, and calculation of the complete energy. The iteration is accepted or rejected based on energy and temperature.

The refined ABCB1, ABCC4 and ABCC5 models were energy minimized using the AMBER 8.0 program package [37]. Two energy minimizations were performed for each model, (1) with restrained backbone by 500 cycles of the steepest descent minimization followed by 500 steps of conjugate gradient minimization, and (2) with no restraints by 1000 cycles of the steepest descent minimization followed by 1500 steps of conjugate gradient minimization. The leaprc.ff03 force field [37], and a 10 Å cut-off radius for non-bonded interactions and a dielectric multiplicative constant of 1.0 for the electrostatic interactions, were used in the molecular mechanics calculations.

The EPS of the ABCB1, ABCC4 and ABCC5 models were calculated with the ICM program, with a potential scale from -10 to +10 kcal/mol.

### Model validation

To check the stereochemical qualities of the ABCB1, ABCC4 and ABCC5 models, the SAVES Metaserver for analyzing and validating protein structures  was used. Programs run were Procheck [44], What_check [45], and Errat [46], and the pdb file of the open inward facing *Escherichia coli *MsbA template [19] was also checked for comparison with the models.

For further validation, the ABCB1, ABCC4 and ABCC5 models were compared with the X-ray crystal structure of the *Mus musculus *ABCB1 [20] and cross-linking and site directed mutagenesis data published on ABCB1 [21-35].

## Results

The 3 ABCB1 models, constructed based on 3 different alignments, where compared with cross-linking data and subsequently also the X-ray crystal structure of the *Mus musculus *ABCB1 [20], and it was revealed that when TMH2 was aligned as the previously published alignments of human ABCB1 and *Escherichia coli *MsbA [19,41], amino acids in TMH2/TMH11 (Val133/Gly939 and Cys127/Ala935) where oriented towards each other in accordance with both cross-linking data and the X-ray crystal structure of the *Mus musculus *ABCB1 [20]. However, when TMH6 was aligned as the previously published alignments of human ABCB1 and *Escherichia coli *MsbA [19,41], ligand binding amino acids (Ile340 and Phe343) pointed away from the drug binding site, while when aligned as proposed from our T-COFFEE [38] alignment, it was in accordance both with cross-linking data and the X-ray crystal structure of the *Mus musculus *ABCB1 [20]. Thus, the ABCB1 model which was most in accordance with cross-linking data and the X-ray crystal structure of the *Mus musculus *ABCB1 [20] was based on the alignment where TMH2 was adjusted according to the previously published alignments of human ABCB1 and *Escherichia coli *MsbA [19,41], while TMH6 was kept exactly as in our T-COFFEE [38] alignment. The alignment of *Escherichia coli *MsbA, human ABCB1 (TMH2 adjusted), human ABCC4 and human ABCC5 used for the homology modelling procedure is shown in Figure 2. For illustrative purposes, only the sequences of the template and the 3 target proteins ABCB1, ABCC4 and ABCC5 are shown.

**Figure 3:**
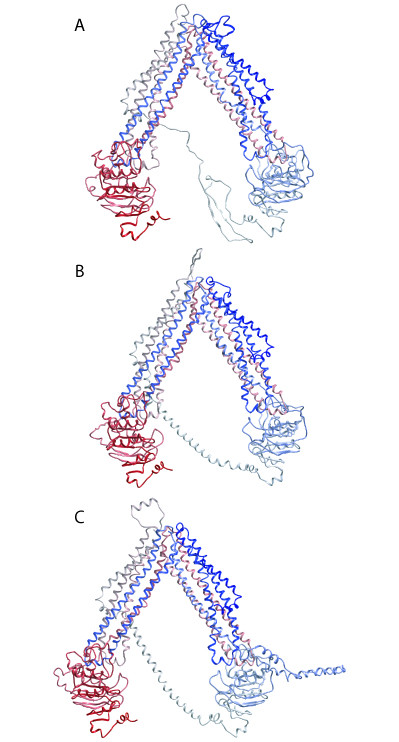
**Backbone Cα-traces of ABCB1 model (A), ABCC4 model (B) and ABCC5 model (C) viewed in the membrane plane, cytoplasm downwards**. Colour coding: blue via white to red from N-terminal to C-terminal.

The energy minimized ABCB1, ABCC4 and ABCC5 models are shown in Figures 3A-C. Each transporter was in an open V-shaped inward conformation with their NBD1 and NBD2 ~50 Å apart. Both Walker A motifs of each model consisted of a coiled loop and a short α-helix (P-loop), and the ATP-binding half sites faced each other. The Walker B motifs were in β-sheet conformation and localized in the NBD's hydrophobic cores, which were constituted of 5 parallel β-sheets. The amino acids localized on the surface of each NBD were mainly charged. In the "arms" of the V-shaped structure, NBD1 was associated with TMHs 1, 2, 3 and 6 (TMD1), and TMHs 10 and 11 (TMD2), while NBD2 was associated with TMHs 4 and 5 (TMD1), and TMHs 7, 8, 9 and 12 (TMD2). Thus, the TMDs were twisted relative to the NBDs, such that TMH4 and TMH5 were crossed over ("cross-over motif" [19]) and associated with TMD2, and TMH10 and TMH11 were crossed over and associated with TMD1. All TMHs contributed to substrate translocation pore, which was closed towards the extracellular side.

**Figure 4:**
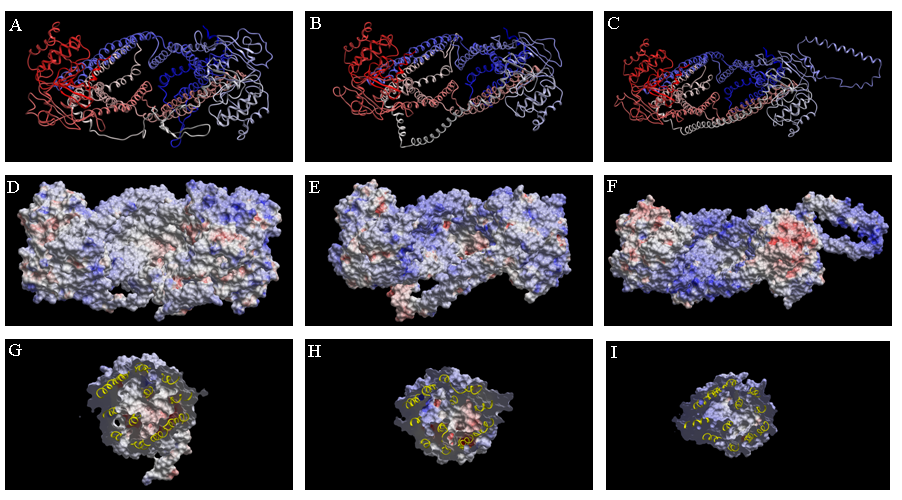
***A-C*: Backbone Cα-traces of ABCB1 model (A), ABCC4 model (B) and ABCC5 model (C) viewed from intracellular side**. Colour coding: blue via white to red from N-terminal to C-terminal. ***D*-*F*:** The water-accessible surfaces of ABCB1 model (D), ABCC4 model (E) and ABCC5 model (F) viewed from intracellular side collared coded according to the electrostatic potentials 1.4 Å outside the surface; negative (-10  kcal/mol), red to positive (+10 kcal/mol), blue. ***G-I***: Cross sections along the inner  membrane layer of water-accessible surfaces of ABCB1 model (G), ABCC4 model (H)  and ABCC5 model (I) viewed from intracellular side, colour coding as D-F. All  illustrations are in similar view.

The loop connecting NBD1 and TMD2 of each transporter was abundant with charged amino acids. The loop connecting NBD1 and TMD2 of ABCB1 was in extended conformation forming a β-sheet between amino acids sections Lys645-Glu652 and Lys665-Ser671, while the loops connecting the subunits of ABCC4 and ABCC5 were α-helical. ABCB5 featured an insertion loop (as compared with the amino acid sequences of *Escherichia coli *MsbA) from Ile479 to His548 in NBD1, and as displayed in Figures 3C and 4C, this loop was pointing away from NBD1 parallel to the membrane. However, modelling loops of lengths as that of the connection between NBD1 and TMD2 is relatively inaccurate and consequently the modelled loop structures must be regarded as uncertain.

Figures 4A-I show the EPS of the substrate recognition area of each of the ABC models. The EPS of the substrate recognition area in the TMDs of ABCB1 was neutral with negative and weakly positive areas, while the EPS of the ABCC5 substrate recognition area was generally positive. The substrate recognition area of ABCC4 was generally positive with negative area "spots".

The results from the stereochemical validations retrieved from the SAVES Metaserver  are shown in Table 1. Overall factors from the Errat option at ~90 indicate that the models were of high quality.

**Table 1:** Results from the stereochemical validations retrieved from the SAVES Metaserver

	**Errat**	**Procheck (%)**				**Whatcheck**
		**Core**	**Allow**	**Gener**	**Disall**	
**ABCB1**	89.7	80.2	15.1	3.4	1.3	Satisfactory
**ABCC4**	86.7	81.1	14.4	3.0	1.5	Satisfactory
**ABCC5**	90.6	80.5	15.1	2.7	1.7	Satisfactory
***Escherichia coli *MsbA **[19]	58.2	54.9	37.4	5.6	2.1	Satisfactory

**Figure 5:**
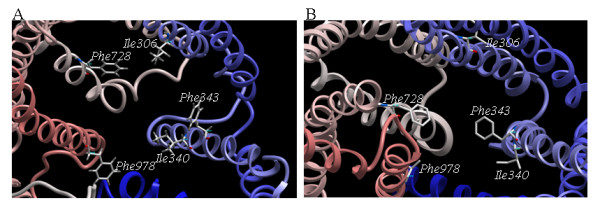
**Comparison of proposed drug binding site in ABCB1 model (A) and the drug binding site in the X-ray crystal structure of P-glycoprotein (ABCB1) **[20]** (B) viewed from the intracellular side with amino acids suggested from site directed mutagenesis studies to take part in ligand binding displayed as sticks coloured according to atom type (C = grey; H = dark grey; O = red; N = blue); Ile306 (TMH5) **[27,35]**, Ile340 (TMH6) **[33]**, Phe343 (TMH6) **[21,27]**, Phe728 (TMH7) **[27]**, and Val982 (TMH12) **[33,35]. Amino acids in panel B are numbered according to human ABCB1. *Mus musculus *numbering: Ile302, Ile336, Phe339 Phe724 and Val978. Differences in helix tilting in the panels refer to the different conformations of ABCB1, outward facing conformation in the left panel and closed conformation in the right panel.

Site directed mutagenesis studies on ABCB1 have indicated that Ile306 (TMH5) [27,35], Ile340 (TMH6) [33], Phe343 (TMH6) [21,27], Phe728 (TMH7) [27], and Val982 (TMH12) [33,35] may participate in ligand binding. As shown in Figure 5A, these residues may form a substrate recognition site in the ABCB1 model. The involvement of these residues in ligand binding is confirmed in the X-ray crystal structure of the *Mus musculus *ABCB1 [20] (Figure 5B). Table 2 shows the corresponding residues in ABCC4 and ABCC5. Measured Cα-Cα distances in the human ABCB1 model, in the X-ray crystal structure of the *Mus musculus *ABCB1 [20] and experimental distance ranges from cross-linking studies and are listed in Table 3.

**Table 2:** Human ABCB1 amino acid residues shown to interact with ligands in site directed mutagenesis studies, corresponding *Mus musculus *ABCB1 amino acids shown to interact with ligand in X-ray crystal structure [20], and corresponding amino acid residues in ABCC4 and ABCC5.

**TMH**	**Human ABCB1**	***Mus musculus *ABCB1 **[20]	**ABCC4**	**ABCC5**
**1**	Leu65 [26]	Leu64	Glu103	Gln190
**5**	Ile306 [27,35]	Ile302*	Ser328	Val410
**6**	Ile340 [33]	Ile336	Gly359	Asn441
**6**	Phe343 [21,27]	Phe339	Arg362	Thr444
**7**	Phe728 [27]	Phe724	Ala727	Ser872
**12**	Val982 [33,35]	Val978	Leu987	Val1137

*) Not direct contact with ligand in *Mus musculus *ABCB1 X-ray crystal structure [20].

**Table 3:** Comparison of Cα-Cα distances in the human ABCB1 model, Cα-Cα distances in the *Mus musculus *ABCB1 X-ray crystal structure [20] and distances between residues from experimental cross-linking studies on ABCB1.

**Region**	**Residues Human ABCB1 (*Mus musculus *ABCB1)**	**Cα-Cα distances (Å)**		**Exp. Cross-linking (Å)**	**Ref**
				
		**Human ABCB1 model**	***Mus musculus *ABCB1 (Pdb code: **3G60)		
TMH1/TMH11	M68/Y950 (M67/Y946)	12.9	9		[25]
	M68/Y953 (M67/Y949)	15.6	10		
	M68/A954 (M67/A950)	17.3	11.2		
	M69/A954 (M68/A950)	19	11.2		
	M69/F957 (M68/F953)	19.5	13		
TMH2/TMH11	V133/G939 (V129/G935)	6.2	5		[24]
	C137/A935 (C133/A931)	7.4	5.1		
TMH4/TMH10	S222/I868 (S218/I864)	34.7	30.3	9-25	[34]
	S222/G872 (S218/G868)	35	30.8		
TMH4/TMH12	L227/S993 (L223/S989)	32.3	22.8	5.5-15	[30]
	V231/S993 (I227/S989)	31.2	20.8		
	W232/S993 (W228/S989)	28.9	20.1		
	A233/S993 (A229/S989)	26	16.3		
	I235/S993 (I231/S989)	30	19.9		
	L236/S993 (L232/S989)	26.6	18		
TMH5/TMH8	N296/G774 (N292/G770)	6.9	8.1		[23]
	I299/F770 (M295/F766)	8.9	9.3		
	I299/G774 (M295/G770)	11.9	10.7		
	G300/F770 (G296/F766)	7.2	7.7		
TMH5/TMH10	I306/I868 (I302/I864)	33	29.9	13-25	[34]
	I306/G872 (I302/G868)	34.3	29.6		
TMH5/TMH11	I306/T945 (I302/T941)	33.4	31.3	13-25	[34]
TMH5/TMH12	I306/V982 (I302/V978)	19	19.8	13-25	[34]
	I306/A985 (I302/A981)	22.4	20.5		
TMH5/TMH12	A295/S993 (A291/S989)	22.7	16.3	5.5-15	[30]
	I299/S993 (M295/S989)	21.4	13.7		
TMH6/TMH7	L339/F728 (L335/F724)	18.8	16.3	20-25	[28]
TMH6/TMH10	P350/V874 (P346/V870)	34.1	23.2	5.5-15	[30]
	P350/E875 (P346/E871)	31.6	20.1		
	P350/M876 (P346/M872)	28.9	18.3		
TMH6/TMH10	L339/I868 (L335/I864)	27	23.4	13-25	[34]
	L339/G872 (L335/G868)	27.9	24		
	L332/Q856 (L328/Q852)	31.9	24.9		
TMH6/TMH11	P350/G939 (P346/G935)	23.5	20.9	5.5-15	[30]
TMH6/TMH11	L339/T945 (L335/T941)	25	22.7	20-25	[32]
TMH6/TMH11	L339/F942 (L335/F938)	24.9	23.9	25	[34]
TMH6/TMH12	L332/L975 (L328/L971)	10.7	12.5	5.5-15	[28]
TMH6/TMH12	F343/M986 (F339/M982)	20.4	15.2		[29]
	G346/G989 (G342/G985)	26.4	15		
	P350/S993 (P346/S989)	31.9	15.2		
TMH6/TMH12	F343/V982 (F339/V978)	18.1	16	10	[28]
	L339/V982 (L335/V978)	17.1	15.9	16-25	
TMH6/TMH12	L339/A985 (L335/A981)	21.2	16.8	20-25	[34]
	L332/L976 (L328/L972)	14	15.6	9-13	
NBD/TMD	L443/S909 (L439/S905)	8.6	12	6-16	[54]
	S474/R905 (S470/R905)	10.1	9.6		
NBD/TMD	A266/F1086 (A262/F1082)	10	9.4	5.5-15	[55]
WalkerA/Signature	S1072/L531 (S1068/L527)	62.4	20.2	5.5-15	[56]
	S1072/S532 (S1068/L528)	59.9	18.5		
	G1073/L531 (G1069/L527)	65	20.6		
	G1073/L532 (G1069/L528)	62.6	19.3		
	G1073/L533 (G1069/L529)	62	20.5		
	C1074/L531 (C1070/L527)	63.4	21.8		
	G1075/L531 (G1071/L527)	64.8	24.9		
	S429/L1176 (S425/L1172)	62.4	25.1		
	G430/L1176 (G426/L1172)	65	25.9		
	C431/L1176 (C427/L1172)	63.3	28.3		
	G432/L1176 (G428/L1172)	64.9	31.9		

Values from experimental cross-linking studies are derived from [41].

## Discussion

Visualization of the molecular structures of human ABC transporters in 3D models contributes to the comprehension of the physical and chemical properties of these molecules, and of their intermolecular interactions with endogenous and exogenous molecules. Thus, interactions involved in determining the potencies and the specificities of different drugs with these drug targets can be identified. To construct a realistic molecular model ("target", e.g. human ABC transporters) by homology, based on an experimental structure ("template", e.g. the open inward facing *Escherichia coli *MsbA [19]), the sequence identity between the target and the template should be relatively high, and the target-template alignment should identify corresponding positions in the target and the template. Homology between two proteins indicates the presence of a common ancestor, and phylogenetic analyses of ABC transporters have indicated that eukaryotic ABCB transporters and ABCC transporters may have originated from bacterial multidrug transporters [47]. It has been shown that the homology modelling approach is at least as applicable to membrane proteins as it is to water-soluble proteins, and that sequence similarities of 30% between template and target will give a Cα-RMSD of 2 Å or less in TMHs [48]. The sequence identities between the template molecule MsbA and the target molecules ABCB1, ABCC4 and ABCC5 are 34%, 21% and 25%, respectively, and the secondary structure elements (NBDs and TMDs) are conserved. Sequence identities between the *Escherichia coli *MsbA TMD and the ABCB1, ABCC4 and ABCC5 TMD1s and TMD2s are 23% (ABCB1-TMD1), 21% (ABCB1-TMD2), 14% (ABCC4-TMD1), 18% (ABCC4-TMD2), 20% (ABCC5-TMD1) and 20% (ABCC5-TMD2), respectively.

A multiple sequence T-COFFEE [38] alignment, which highlights evolutionary relationships and increases probability that corresponding sequence positions are correctly aligned, was used to create the target-template alignments in this study. The T-COFFEE [38] alignment differed from the previously published alignments of human ABCB1 and *Escherichia coli *MsbA [19,41] in TMH2 and TMH6. The ABCB1 model based on the combined alignment, with TMH2 adjusted corresponding to the previously published alignments of human ABCB1 and *Escherichia coli *MsbA [19,41], was in the best agreement with cross-linking data and the X-ray crystal structure of the *Mus musculus *ABCB1 [20]. This illustrates that combining different alignment methods may strengthen the alignment used for homology modelling. The alignment correctly aligning TMH2 was created using ClustalW and HMMTOP [41], while T-COFFEE, which aligned TMH6 correctly, is broadly based on progressive approach to multiple alignment using a combination of local (Lalign) and global (ClustalW) pair-wise alignments to generate a library of alignment information which is used to guide the progressive alignment.

The X-ray crystal structure of the *Mus musculus *ABCB1 [20] and site directed mutagenesis studies on ABCB1 may serve as validity tests both for helix orientation in the template [19], and for the alignment used for ABC transporter modelling (Figure 2). The helix orientation of the 12 TMHs of the ABCB1 model was in accordance with the X-ray crystal structure of the *Mus musculus *ABCB1 [20]. Both the ABCB1 model and the ABCB1 X-ray structure exhibited a V-shaped structure with the same relative domain orientations; TMDs twisted relative to the NBDs with TMH4 and TMH5 crossed over ("cross-over motif" [19]) and associated with TMD2, and TMH10 and TMH11 crossed over and associated with TMD1. The major difference between the ABCB1 model and the X-ray crystal structure of the *Mus musculus *ABCB1 [20] was that the V-shape of the ABCB1 model was wider than the X-ray crystal structure of the *Mus musculus *ABCB1 [20].

Cα-Cα distances in the human ABCB1 model, Cα-Cα distances in the *Mus musculus *ABCB1 X-ray crystal structure [20], and distances between residues in the TMD area from experimental cross-linking studies on ABCB1, are listed in Table 3. As shown in the table, the Cα-Cα distances in the human ABCB1 model compared to the Cα-Cα distances in the *Mus musculus *ABCB1 X-ray crystal structure [20] revealed that the helix packing of TMH pairs 2 and 11, 5 and 11, 6 and 7, and 6 and 11, were only 1-2 Å further apart in the human ABCB1 model. TMHs 5 and 8 were packed approximately 1 Å tighter in the human ABCB1 model than in the *Mus musculus *ABCB1 X-ray crystal structure [20]. TMH pairs 1 and 11, 4 and 10, and 5 and 10 were approximately 3-7 Å further apart in the human ABCB1 model than in the *Mus musculus *ABCB1 X-ray crystal structure [20]. The most striking differences between helix packing of the human ABCB1 model and the *Mus musculus *ABCB1 X-ray crystal structure [20] were observed in TMHs 6 and 12. Whereas the differences of their packing towards other TMHs where in the range of 1-5 Å towards the extracellular side, the differences between the distances between these TMHs long in the human ABCB1 model and the *Mus musculus *ABCB1 X-ray crystal structure [20] were up to 15 Å towards the cytoplasm. This indicates that in order for ABCB1 to attain a wide open inward facing conformation, large conformational changes involving a scissors like movement of TMH6 and TMH12 may take place.

As shown in Figure 5A, Ile306 (TMH5) [27,35], Ile340 (TMH6) [33], Phe343 (TMH6) [21,27], Phe728 (TMH7) [27], and Val982 (TMH12) [33,35] may form a substrate recognition site in the ABCB1 model. The involvement of these amino acid residues is also confirmed by the X-ray crystal structure of the *Mus musculus *ABCB1 [20]. Interestingly, Ile306 (Ile302 in *Mus musculus *ABCB1) actually points slightly towards the membrane in the X-ray crystal structure, while it points directly towards the translocation pore in the ABCB1 model (Figure 5). This could be due to twisting of TMH5 upon changing conformation from at drug recognition conformation to a drug bound conformation. Cross-linking studies on ABCB1 has proposed that residue pairs Asn296-Gly774, Ile299-Phe770, Ile299-Gly774, and Gly300-Phe770 (TMH5 and TMH8, respectively), are adjacent [23]. These residues are in direct contact with each other in the ABCB1 model presented in this study. Furthermore, cross-linking studies has also shown that Val133 and Cys137 (TMH2) are close to Ala935 and Gly939 (TMH11) [24]. In the present ABCB1 model, these residues are adjacent. This also implies that the orientations of these residues in the models are correctly localized, and that the alignment used for the ICM homology modelling procedure is correct.

As shown in Table 3, the Cα-Cα distances in the human ABCB1 model of residues that connect residues on both sides of the wings are substantially longer than distances measured by chemical cross-linking. This may be due to drug-induced fit in the cross-linking experiments, which is not reflected in the present open inward ABCB1 model. Interestingly, the corresponding Cα-Cα distances in the *Mus musculus *ABCB1 X-ray crystal structure [20] are also longer than distances measured by chemical cross-linking. The shorter distances measured by chemical cross-linking may represent conformations of ABCB1 that are closed to the cytoplasmic side, with the wings tighter than in the conformations of the human ABCB1 model and the *Mus musculus *ABCB1 X-ray crystal structure [20].

The open inward facing *Escherichia coli *MsbA template may represent a functional inward-facing conformation of the transporter, even though conformational disruption of the protein due to the presence of detergent molecules during crystallization cannot be excluded. According to the Errat option of the SAVES Metaserver for analyzing and validating protein structures, which indicated that the stereochemical qualities of the models were realistic, the stereochemical quality of the template was poorer than the stereochemical qualities of the ABC transporter models (Table 1). This difference in quality may be due to the modelling procedures; the ABC transporter models were energy minimized using the AMBER 8.0 program package [37], whereas the template was not.

Several ABCB1 models have previously been published [49-52] based on an MsbA X-ray crystal structure that was subsequently retracted [53]. In 2009, 4 molecular models of human P-glycoprotein in two different catalytic states were published [41] based on X-ray crystal structures of the bacterial MsbA in different conformations [19]. These models are based on the previous alignments of human ABCB1 and *Escherichia coli *MsbA [19,41], and consequently, the orientation of their TMH6 differ from the orientation of TMH6 in the ABCB1 model presented in this study. The measured Cα-Cα distances in our present ABCB1 model are in accordance with the corresponding distances in their open inward ABCB1 model [41].

**Figure 6:**
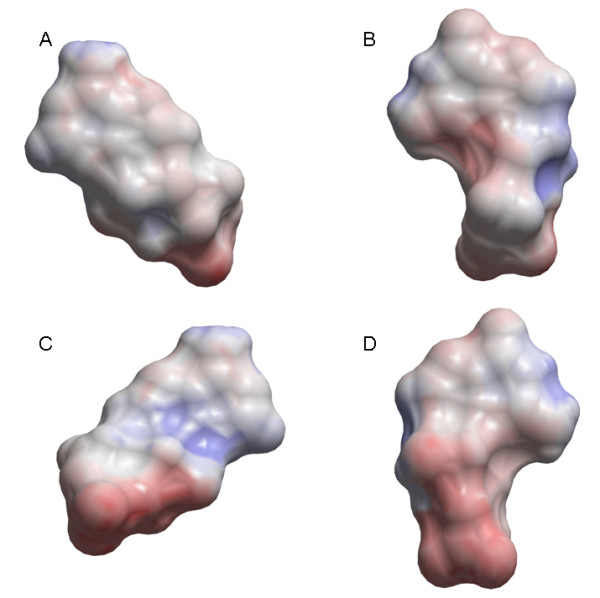
**The surface of cAMP (panel A and C) and cGMP (panel B and D) colour coded according to electrostatic potentials outside the surface**. The surface of cAMP in panel C are flipped 180° along the y-axis relative to panel A, while the surface of cGMP in panel D are flipped 180° along the y-axis compared with panel B.

From a pharmacological point of view, the EPS of the ligand recognition area in the wide open conformation of each of the ABC transporters is of particular interest, since it may elucidate substrate differences between these transporters. The template structure was constructed by fitting the X-ray structure of outward facing MsbA to the electron density map of inward facing MsbA. The template conformation may therefore have limitations that can affect the calculated EPS in some regions of the models. ABCB1 transports cationic amphiphilic and lipophilic substrates [5-8], and, as illustrated in Figure 4, the EPS of its ligand recognition area was neutral with negative and weakly positive areas. In contrast, ABCC4 and ABCC5 transport organic anions [9], and the EPS of the ABCC5 substrate recognition area was generally positive. Interestingly, the substrate recognition area of ABCC4 was generally positive with negative area "spots". This may raise reflections over differences in substrate selectivity between the anionic transporters ABCC4 and ABCC5, and support the reports of ABCC4 with preference for cAMP and ABCC5 with preference for cGMP [9,10]. The EPS of cAMP and cGMP (Figure 6) indicates that the surface of cGMP (Figure 6, panel B and D) has a larger region of negative EPS than that of cAMP (Figure 6, panel A and C). This may indicate that cGMP binds stronger to the surface of ABCC5 than cAMP, while negative area "spots" on the surface of ABCC4 may contribute to stronger binding to cAMP than to cGMP.

The residues of the binding site of the ligand bound *Mus musculus *ABCB1 X-ray crystal structure [20] and the respective binding site of all three models are shown in Table 2. While the binding sites of human and *Mus musculus *ABCB1 features lipophilic residues (Leucine, isoleucines, phenyl alanines, valine), ABCC4 has charged and polar residues and ABCC5 has polar residues. A positively charged residue in the binding site area of ABCC5, Lys448, also may take part in interaction with organic anions. The binding sites of the ABCB1, ABCC4 and ABCC5 models are wider and more accessible to the cytoplasm than the binding site of the *Mus musculus *ABCB1 X-ray crystal structure [20], reflecting their wide open-inward conformation.

Crystal structures of ABC transporters captured in different conformations have revealed that ABC transporter mechanism involves alternating access of substrate from the inward to the outward facing conformation, with subunit twisting and domain swapping [18-20]. The putative substrate recognition pocket in the ABCB1, ABCC4 and ABCC5 models in the wide open inward conformation presented in this study contains the same amino acid residues as the putative substrate releasing pocket in our previous outward-facing molecular models of ABCB1 [15], ABCC4 [16] and ABCC5 [17] based on the *Staphylococcus aureus *ABC transporter Sav1866 [18]; Ile306 (TMH5) [27,35], Ile340 (TMH6) [33], Phe343 (TMH6) [21,27], Phe728 (TMH7) [27], and Val982 (TMH12) [33,35]. This indicates that these residues contribute to a substrate translocation pore that changes conformation from a high affinity inward facing substrate recognition binding site to a low affinity outward facing substrate releasing pocket. Mutating the corresponding residues of ABCC4 and ABCC5 (Table 2) into the ABCB1 residues would be a valuable test of our models. The models indicate that these mutants may have substrate specificity more similar to that of wild type ABCB1. Leu65 (TMH1) [26], which is also suggested to take part in ligand binding, and is localized in the substrate releasing pocket in the outward facing ABCB1 model [15], is slightly distant from the core area of the ligand recognition site in the inward facing ABCB1 model. This amino acid may come into contact with the ligand upon conformational changes associated with ligand binding.

The models presented in this study may represent a substrate recognition conformation, and from a structure aided drug design point of view, the specificity and affinity of ABC transporter substrate binding in this conformation is of particular interest. When performing docking studies, the structural flexibility of transporters, and the structural changes of the drug and the drug target adopting an energetically favourable complex (induced-fit), as has been demonstrated in a cysteine-scanning mutagenesis and oxidative cross-linking study of substrate-induced changes in ABCB1 [22], should be considered in order to predict how a designed drug will fit into the drug target.

The ABCB1, ABCC4 and ABCC5 models presented in this study should be considered as working tools for generating hypotheses and designing further experimental studies related to ABC transporter structure and function, and their drug interactions. The binding site of the ABCB1 transporter model is in accordance with X-ray crystal structure of the *Mus musculus *ABCB1 [20] and site directed mutagenesis data and cross-linking studies on ABCB1 [21-35], indicating that the open inward-facing conformation structure of *Escherichia coli *MsbA [19] is a suitable template for homology modelling of ABCB1, ABCC4 and ABCC5. The corresponding residues in ABCC4 and ABCC5 (Table 2) are candidates for point mutations in site directed mutagenesis studies.

Co-ordinates of the ABCB1, ABCC4 and ABCC5 models are available from the authors upon request.

## Competing interests

The authors declare that they have no competing interests.

## Authors' contributions

AWR carried out the molecular modelling studies (homology modelling and model refinement, and quality validation), created sequence alignments, and drafted the manuscript. IS participated in the design of the study, and contributed with bioinformatics advice and critical review of the manuscript. GS conceived of the study and participated in its design, contributed with biological advice and critical review of the manuscript. All authors read and approved the final manuscript.
